# Massive Digestive Hemorrhagia Revealing a Gastro-Intestinal Stromal Tumor of the Jejunum

**DOI:** 10.7759/cureus.17316

**Published:** 2021-08-20

**Authors:** Yasmine Cherouaqi, Fatima zahra Belabbes, Hanane Delsa, Anass Nadi, Fedoua Rouibaa

**Affiliations:** 1 Gastroenterology and Proctology, Faculty of Medicine, Mohammed VI University of Health Sciences (UM6SS) Cheikh Khalifa International University Hospital, Casablanca, MAR

**Keywords:** jejunum, melena, hemorrhagia, c-kit, imatinib, gastrointestinal stromal tumor

## Abstract

Gastrointestinal stromal tumors (GISTs) are mesenchymal tumors that originate from Cajal cells located in different sites of the digestive system. They may occur in the entire gastrointestinal tract. They are diagnosed on the basis of the identification of c-kit-positive cells. We report a case of a stromal tumor of the jejunum revealed by a massive digestive hemorrhagia. Surgical resection is the basis of the treatment of GISTs. Imatinib, a tyrosine kinase inhibitor, is a beneficial treatment after surgical resection of high-risk GISTs.

## Introduction

Gastrointestinal stromal tumors (GISTs) are the most common mesenchymal tumors of the gastrointestinal (GI) tract. They may occur in the entire length of the GI system, from the esophagus to the anus, and sometimes in the omentum [1]. The small intestine is the second most frequent location of GISTs after the stomach. The jejunal GISTs account for only 0.1% to 3% of all gastrointestinal tumors [2]. GISTs are diagnosed on the basis of the identification of c-KIT-positive cells [1]. In addition to surgery, they can be treated by imatinib which inhibits the KIT kinase activity. The treatment of advanced GIST is evolving rapidly with the development of new molecular compounds such as avapritinib and ripretinib [3]. In this work, we present an unusual case of a jejunal GIST revealed by a massive hemorrhage.

## Case presentation

**Figure 1:**
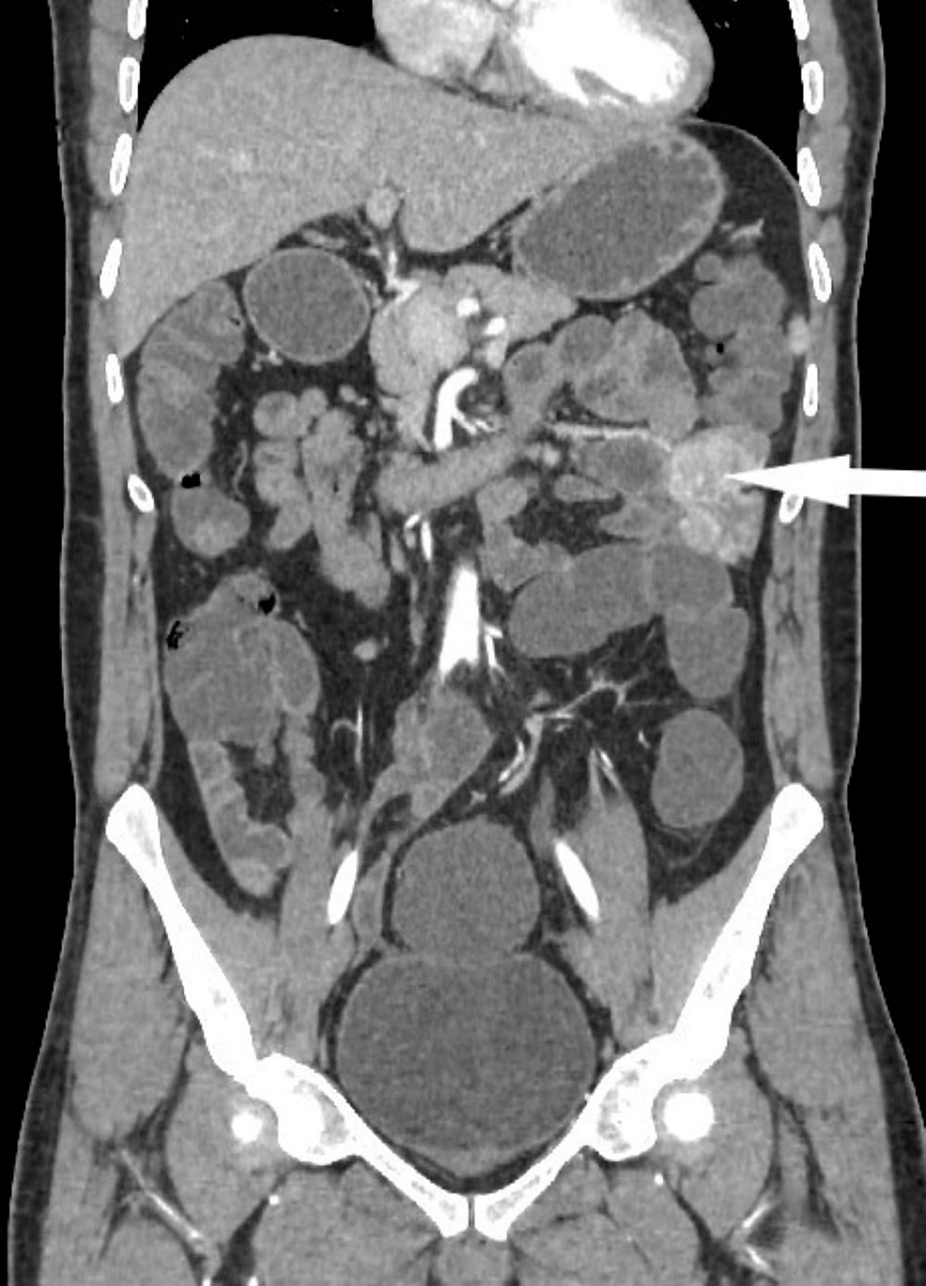
The entero-CT showing the jejunal mass

A 40-year-old male with no specific pathological history presented high volume melena. He had several episodes of melena for the last six years, periumbilical pain, vomiting, a not-tolerated anemic syndrome, a weight loss of nine kilograms, and asthenia. He didn’t have hematemesis, hematochezia, transit disorder, or anorexia. The physical examination at the admission found a tachycardia patient at 110 beats per minute, a normal blood pressure, oxygen saturation of 96%, and discolored conjunctiva. The abdominal exam was normal, as well as the rest of the clinical examination. At the biology report, there was normochromic normocytic anemia at 8.9 g/dL. The rest of the biological examination was normal. After transfusion and hemodynamic stabilization, the patient had an oeso-gastroduodenal fibroscopy. It showed erythematous gastritis. The ileocolonoscopy showed melena without any endoscopic lesion. An entero-computed tomography (CT) revealed a parietal tumor mass of the third jejunal loop, with endoluminal development and irregular outlines, measuring 34x30 mm, extended over 5 cm, with infiltration of the adjacent fat. This mass was in contact with the adjacent jejunal loop without separation of the border (Figure 1). The thoracic CT showed a pleural nodule with a non-specific appearance.

Surgical intervention was indicated for therapeutic purpose. The exploration of the entire abdominal cavity didn’t find any metastases or lymph node extension (Figue 2). The patient had a segmental enterectomy of 15 cm of the jejunal GIST with an end-to-end jejunal anastomosis (Figure 3). There were no complications in the postoperative period. The histopathologic exam showed a gastrointestinal stromal tumor with a low risk of recurrence. The mitotic index was less than five. The patient started imatinib with good tolerance. The abdominal CT after six months, one year, and two years of treatment were normal.

**Figure 2:**
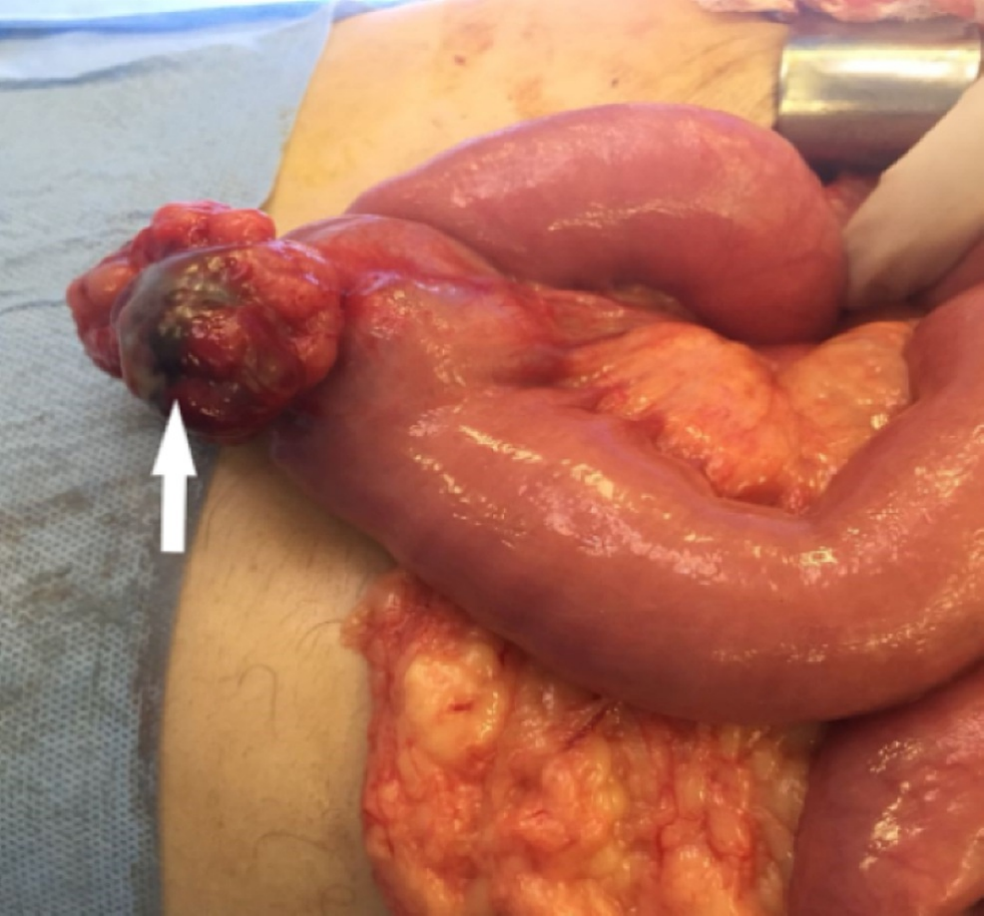
The appearance of the jejunal GIST during surgery GIST - gastrointestinal stromal tumor

**Figure 3:**
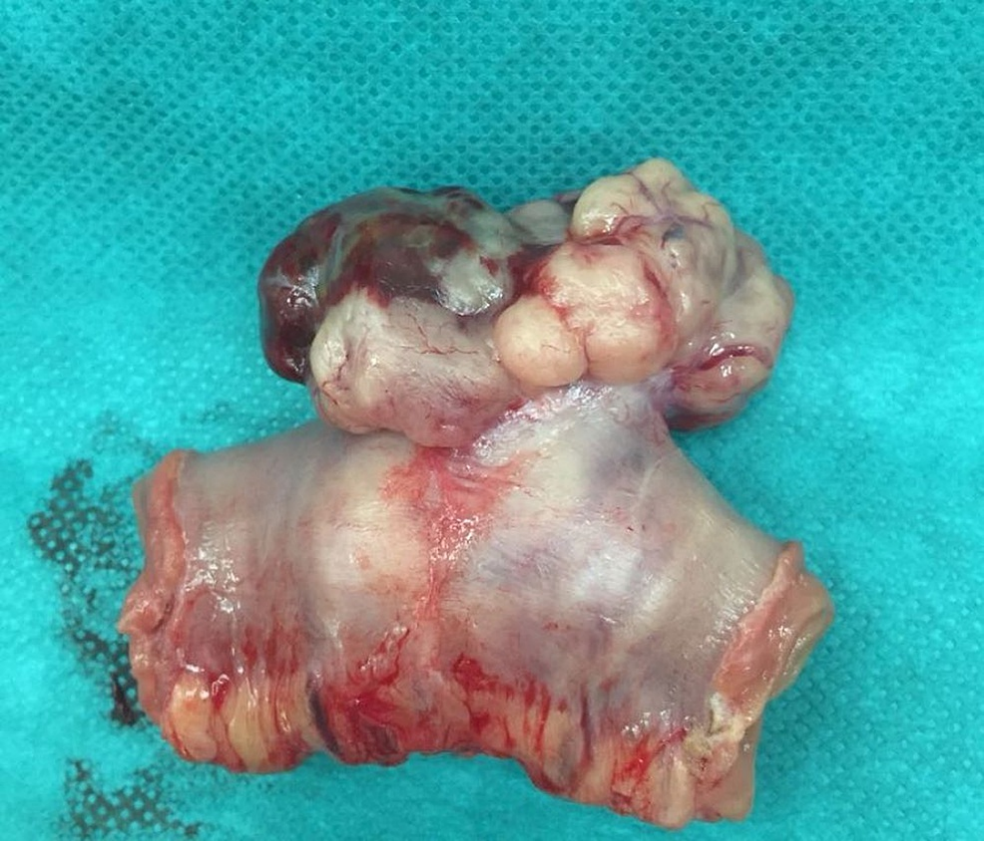
The jejunal GIST after surgical resection GIST - gastrointestinal stromal tumor

## Discussion

Gastrointestinal stromal tumors are specific tumors of the gastrointestinal tract. They originate from Cajal cells located in different sites of the digestive tract [4]. They are a rare group of digestive tumors that make up about one percent of all gastrointestinal cancers [5]. In the stomach, the most common site of GIST, benign tumors outnumber the malignant ones by a wide margin, whereas most esophageal and colonic GISTs are malignant. In the early literature, these tumors were classified as leiomyomas, cellular leiomyomas, leiomyoblastomas, and leiomyosarcomas [6]. They are defined as KIT-positive (CD17, stem cell factor receptor) mesenchymal spindle cell neoplasm or primary epithelioid neoplasm in the gastrointestinal tract, omentum, and mesentery [6]. GISTs have a peak incidence between the 5th and 6th decades, although it may occur at any age [7]. They are very rare in children [8]. There is no predilection for both sexes [7]. Only 70% of patients with a GIST are symptomatic. In 20% of cases, they are asymptomatic, and tumors are detected incidentally. The symptoms are not specific to the disease [9-10]. The clinical presentation of patients with GISTs depends on the anatomic location of the primary lesion. The most frequent symptoms are abdominal pain (67.4%), abdominal mass (31.2%), bowel obstruction (24.1%), hematochezia (21.3%), and fever (14.2%). They can be responsible for chronic gastrointestinal bleeding, causing hematemesis, melena, or anemia. In rare cases, acute massive bleeding can occur [11]. Wild-type GIST is frequently associated with inherited syndromes such as carney's triad and neurofibromatosis type 1. They often affect the pediatric population and young adults [12]. The preoperative diagnosis can be difficult. It is often confirmed during surgery. Endoscopic and imaging examinations are often essential to establish the preoperative diagnosis. The study of the small intestine requires the use of entero-magnetic resonance imaging (MRI). It is an alternative to entero-CT. The GISTs most often have the appearance of an exophytic mass with regular contours. They can also take on the appearance of a small wall nodule as in CT. In T1 weighting, the signal from GISTs is generally intermediate, sometimes with hypersignals indicating hemorrhage [13]. The endoscopic appearance of GISTs is not very specific. It is generally an aspect of a regular nodule, submucosal in appearance because it is covered with normal mucosa. Endoscopic biopsies are most often negative. Ultrasound endoscopy is the best examination to characterize eso-gastro-duodenal or rectal submucosal lesions. GISTs appear as inhomogeneous hypoechoic lesions [14]. Endoscopic, endosonographic, or imaging features are suggestive but not diagnostic of GIST. In practice, only the anatomopathological aspect, and especially the search for the expression of c-KIT, will allow the diagnosis to be confirmed with certainty. Histologically, GISTs are of three types: spindle cell type (70%), epithelial type (20%), and mixed type (10%). The immunohistochemical study shows the expression of KIT by tumor cells in 95% of cases [15]. The National Institutes of Health (NIH) and Armed Forces Institute of Pathology (AFIP) risk classification criteria are commonly used to predict the prognosis of GISTs. The aggressiveness of GISTs depends on size, mitotic index, and location [16-17]. Metastases in GISTs have been reported in 50% of patients. The vast majority of metastases from GISTs, at presentation or at disease recurrence, are intra-abdominal, with metastases to the liver, omentum, or peritoneal cavity [18]. Treatment of GISTs depends on risk stratification. Surgery is the treatment of choice, but various endoscopic resection modalities are being more and more tried. Surgery is the treatment of choice for symptomatic localized GIST [18-19]. Endoscopic therapy is mainly used for the treatment of GISTs of the upper digestive tract in patients without recurrence or metastasis [19]. The tyrosine kinase inhibitor (TKI), imatinib, radically changed the natural history of KIT-induced GISTs; approximately 90% of GISTs have an activating mutation of the KIT or PDGFR alpha oncogene known to confer sensitivity to imatinib [20]. Approximately 80% of patients with metastatic GIST show at least some clinical response to imatinib, a targeted small molecule KIT inhibitor [3].

## Conclusions

GISTs are rare tumors in our context. Testing for KIT and PDGFRA gene mutations is recommended, with exception of GISTs of very low risk of recurrence. Imatinib is the standard adjuvant treatment after R0 resection of GIST of high risk of recurrence and the first-line treatment of advanced GIST. This case presents an unusual presentation of a jejunal GIST and its management. More researches are necessary to better clarify and update their management.
